# Application of machine learning in the prediction of COVID-19 daily new cases: A scoping review

**DOI:** 10.1016/j.heliyon.2021.e08143

**Published:** 2021-10-11

**Authors:** Soudeh Ghafouri-Fard, Hossein Mohammad-Rahimi, Parisa Motie, Mohammad A.S. Minabi, Mohammad Taheri, Saeedeh Nateghinia

**Affiliations:** a Department of Medical Genetics, Shahid Beheshti University of Medical Sciences, Tehran, Iran; b Dental Research Center, Research Institute of Dental Science, Shahid Beheshti University of Medical Sciences, Tehran, Iran; c Sirjan University of Technology, Kerman, Iran; d Urology and Nephrology Research Center, Shahid Beheshti University of Medical Sciences, Tehran, Iran; e Skull Base Research Center, Loghman Hakim Hospital, Shahid Beheshti University of Medical Sciences, Tehran, Iran

**Keywords:** COVID-19, Machine learning, Artificial intelligence, Spread, Global pandemic

## Abstract

COVID-19 has produced a global pandemic affecting all over of the world. Prediction of the rate of COVID-19 spread and modeling of its course have critical impact on both health system and policy makers. Indeed, policy making depends on judgments formed by the prediction models to propose new strategies and to measure the efficiency of the imposed policies. Based on the nonlinear and complex nature of this disorder and difficulties in estimation of virus transmission features using traditional epidemic models, artificial intelligence methods have been applied for prediction of its spread. Based on the importance of machine and deep learning approaches in the estimation of COVID-19 spreading trend, in the present study, we review studies which used these strategies to predict the number of new cases of COVID-19. Adaptive neuro-fuzzy inference system, long short-term memory, recurrent neural network and multilayer perceptron are among the mostly used strategies in this regard. We compared the performance of several machine learning methods in prediction of COVID-19 spread. Root means squared error (RMSE), mean absolute error (MAE), R^2^ coefficient of determination (R^2^), and mean absolute percentage error (MAPE) parameters were selected as performance measures for comparison of the accuracy of models. R^2^ values have ranged from 0.64 to 1 for artificial neural network (ANN) and Bidirectional long short-term memory (LSTM), respectively. Adaptive neuro-fuzzy inference system (ANFIS), Autoregressive Integrated Moving Average (ARIMA) and Multilayer perceptron (MLP) have also have R^2^ values near 1. ARIMA and LSTM had the highest MAPE values. Collectively, these models are capable of identification of learning parameters that affect dissimilarities in COVID-19 spread across various regions or populations, combining numerous intervention methods and implementing what-if scenarios by integrating data from diseases having analogous trends with COVID-19. Therefore, application of these methods would help in precise policy making to design the most appropriate interventions and avoid non-efficient restrictions.

## 1 Introduction

**Figure 1:**
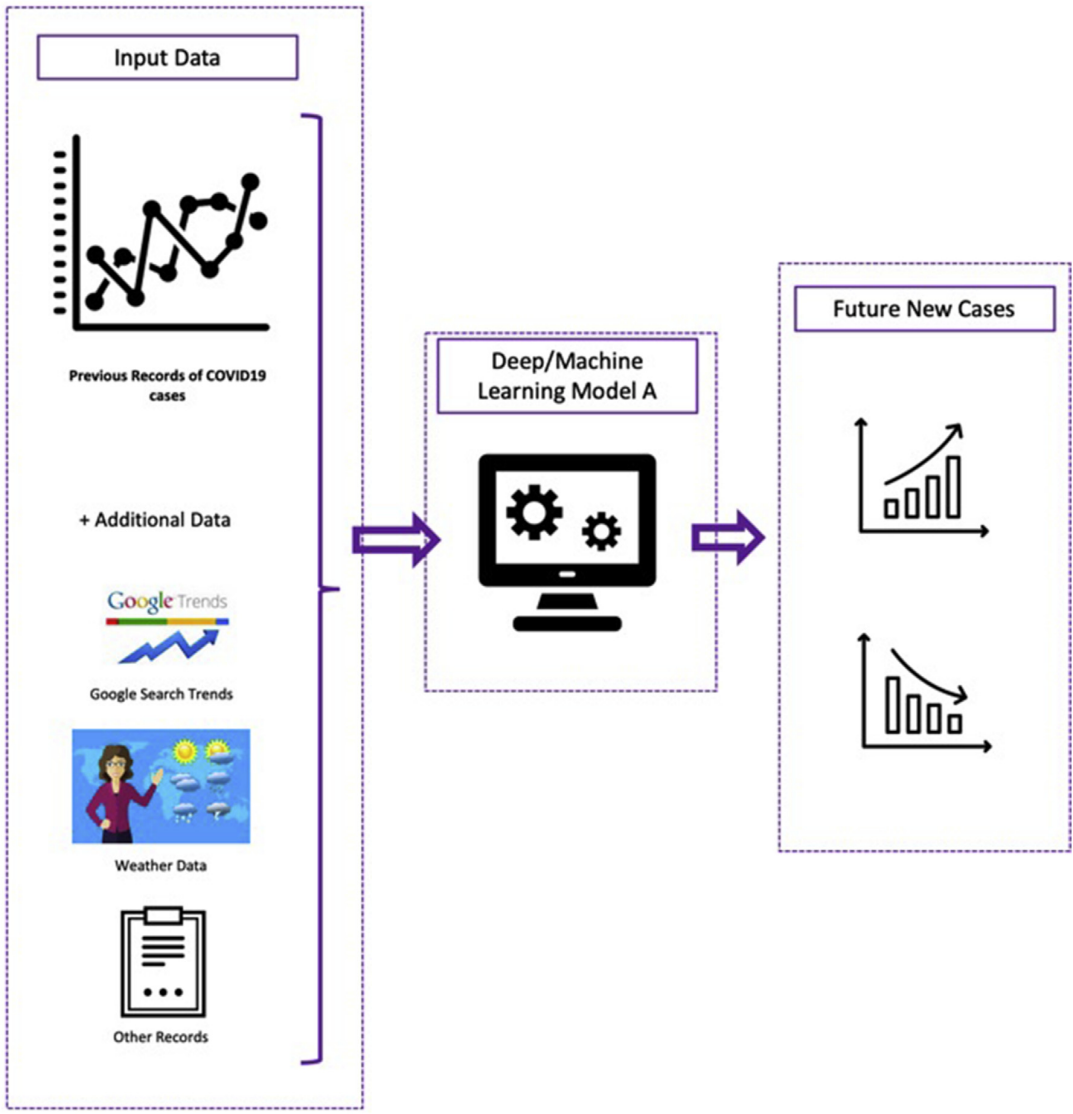
The role of artificial intelligence approaches for prediction of COVID-19 spread.

The novel coronavirus disease initiated in the late 2019 (COVID-19) is resulted from the infection with the severe acute respiratory syndrome-coronavirus 2 (SARS-CoV-2) [1]. Since late 2019, it has spread globally, leading to a persistent pandemic. COVID-19 spread is dependent on inter-individual close contacts and transmission of breath droplets. Prediction of the rate of COVID-19 spread and modeling of its course have critical impact not only for health systems but also for policy makers. In fact, policy making relies on discernments formed by prediction models to propose new strategies and to measure the efficiency of the imposed policies. Based on the nonlinear and complex nature of this disorder [2] application of artificial intelligence methods is an appropriate alternative to traditional epidemic models for prediction of its spread. Although some traditional epidemic models such as Susceptible-Exposed-Infective-Recovery has been used for prediction of epidemic course [3], these methods have some limitations. For instance, the validity of the Susceptible-Exposed-Infective-Recovery model relies on precise appraisal of virus transmission features including the basic reproductive quantity R_0_ as well as incubation and infectious periods which are rather difficult to be estimated in real contexts [4]. Figure 1 shows the role of artificial intelligence approaches for prediction of COVID-19 spread.

Machine learning methods usually use data sequences retrieved over a period of time as inputs to predict course of COVID-19 epidemic. Several strategies have been implemented for prediction of COVID-19 spread. Among the applied strategies is the Long short-term memory (LSTM) model. For instance, Multilayer perceptron (MLP) has also been applied for modeling of COVID-19 spread. This method has facilitated prediction of the highest number of persons who are affected by COVID-19, the highest number of people who recovered, and the highest number of mortalities per place in each time division [5]. LSTM with the Natural language processing (NLP) module has been used to assess the infection frequency and enhance the predictive accuracy of the model [6]. LSTM can efficiently improve gradient explosion and gradient disappearance in the course of the training process by presenting the constant error carousel unit [6]. LSTM is superior to the traditional Recurrent neural network (RNN) in term of its good enactment in apprehending the long-term dependency of sequences, thus being appropriate for the categorization, processing, and forecasting the long sequence data [7]. Based on the importance of machine and deep learning methods in the prediction of COVID-19 spreading trend, in the current study, we reviewed studies which used these strategies to envisage the number of new cases of COVID-19. The research question was: “What are the applications of machine learning systems and their performances in the prediction of COVID-19 daily new cases?“. In the current study we were looking for publications that evaluate the performance of any machine learning or deep learning approaches based on the research question inclusion and exclusion criteria.

The following parameters were extracted: Root means squared error (RMSE), Mean absolute error (MAE), R^2^ coefficient of determination (R^2^), and Mean absolute percentage error (MAPE). These parameters are the main parameters which are applied to assess the error rates of forecasting and performance of the model in regression analysis. MAPE is calculated based on percentage errors.

## 2 Materials and methods

We used PRISMA Scoping review guidelines and checklist.

### 2.1 Protocol

Reporting this scoping review is based on Preferred Reporting Items for Systematic Reviews and Meta- Analyses extension for scoping reviews [8].

### 2.2 Exclusion criteria

1) Studies that did not report or evaluate their prediction regarding the daily confirmed cases or cumulative number of confirmed cases.
2) Studies that did not report at least one of the Root means squared error (RMSE), Mean absolute error (MAE), R^2^ coefficient of determination (R^2^), and Mean absolute percentage error (MAPE) in their measurements.

### 2.3 Information sources and search

An electronic search was conducted in PubMed, Google Scholar, Scopus, Embase, arXiv, and medRxiv for finding the relevant literature from January 2020, to June 2021. Different combinations of the following keywords were used in the search procedure: “machine learning”, “deep learning”, “neural network”, “artificial intelligence”, “Covid-19”, “incidence”, “prevalence”, “spread∗“, “new cases”, “predict∗“, and “forecast∗“.

### 2.4 Selection of sources of evidence

**Figure 2:**
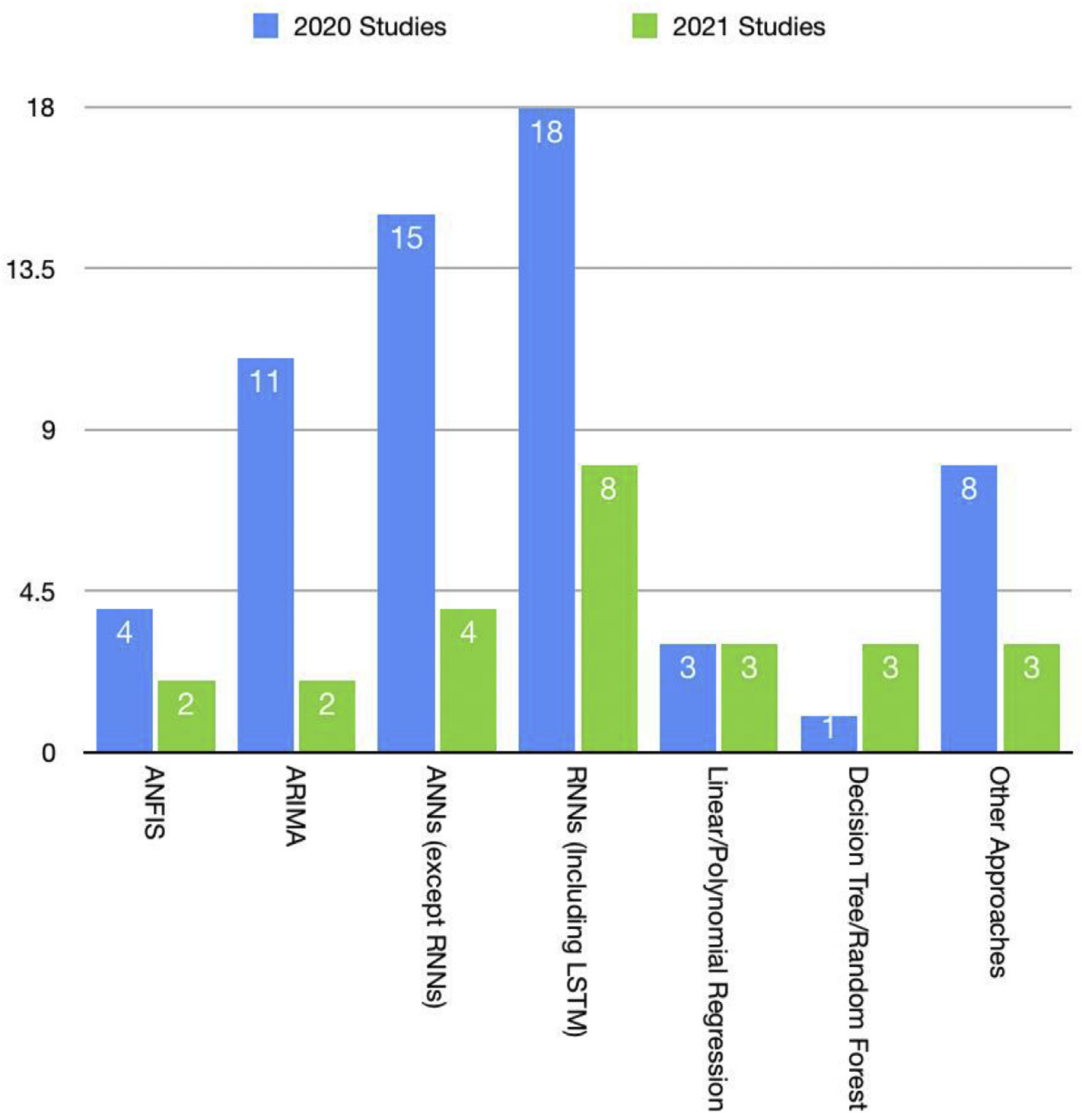
The flowchart of the protocol of systematic literature review.

Duplicate studies were removed. Studies that were cited within the retrieved papers were reviewed for finding any missing studies. For identifying the proper journal papers and conference proceedings, our team members screened the title and abstracts based on inclusion and exclusion criteria independently. Finally, considering the inclusion and exclusion criteria, investigators identified the eligible publications in this stage independently. Figure 2 illustrates the flowchart of the protocol of systematic literature review.

### 2.5 Data charting process

Two investigators were responsible for extracting the data, separately. The charting process was followed by consensus to resolve any disagreements.

### 2.6 Data items

For the selected studies, the following data have been extracted: regions (e.g., countries, states, etc), data source, data structure, machine learning model and model performance including RMSE, MAE, R^2^, MAPE (on the basis of the best model). These performance measures were selected, since they are the most common performance measurement among the selected studies.

## 3 Results

**Figure 3:**
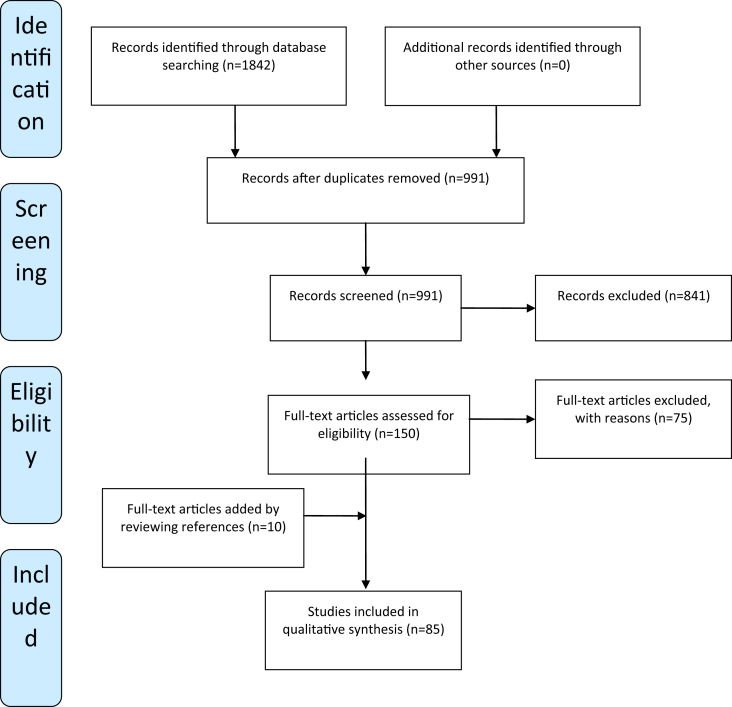
The number of included studies used each machine learning approaches (In two studies, more than one type of models were considered).

Several artificial intelligence strategies have been used for prediction of COVID-19 spread using different models (Figure 3).

### 3.1 Adaptive neuro-fuzzy inference system (ANFIS)

ANFIS is a type of artificial neural network being founded on Takagi–Sugeno fuzzy inference system. Architecture of ANFIS has five layers, namely fuzzification layer, the layer which generates the firing strengths for the rules (rule layer), the layer that normalizes the computed firing strengths, the layer which receives as input the normalized values and the consequence parameters, and the layer that returns the final output [9]. Al-Qanes et al. [10] have designed an upgraded kind of the ANFIS model to estimate the quantity of infected persons in four countries, namely Italy, Iran, Korea, and the USA. Their model has been founded on a novel nature-inspired optimizer, namely the marine predators algorithm (MPA). This algorithm has optimized the ANFIS variables, increasing its predicting performance. They have shown superiority of the MPA-ANFIS method to previously suggested predicting models in terms of better values for RMSE, MAE, MAPE, and R^2^ [10]. In another study, ANFIS was boosted using an improved flower pollination algorithm (FPA) by using the salp swarm algorithm (SSA). The suggested FPASSA-ANFIS model was then appraised using the official data retrieved from WHO site. Moreover, the accuracy of the suggested model was then appraised using two distinct datasets of weekly influenza cases [11]. Alsayed et al. [12] have predicted the epidemic peak in Malaysia using the Susceptible-Exposed-Infectious-Recovered (SEIR) model. They have also used the ANFIS model short-time prediction of the amount of infected individuals. They have also demonstrated the impact of interventions on postponing the epidemic peak. Moreover, they have suggested that extension of the intervention period might decrease the epidemic magnitude at the peak. This study has reported RMSE, R^2^ and MAPE values as 46.87, 0.9973 and 2.79, respectively [12]. Thus, this study has reported the best performance measurements using this method. Behnood et al. [13] have used an integration of the virus optimization algorithm (VOA) and ANFIS to appraise the impact of numerous climate-associated parameters and population density on COVID-19 spread. They have demonstrated the remarkable influence of population density on the performance of their designed models, emphasizing on the prominence of social distancing in decreasing COVID-19 infection rate and spread. RMSE, MAE and R^2^ values have been reported to be 22.47, 7.33 and 0.83, respectively [13].

### 3.2 Autoregressive Integrated Moving Average (ARIMA)

As a type of univariate regression analysis method, ARIMA forecasts upcoming values according to differences between values instead of actual figures. As a generalization of an autoregressive moving average (ARMA) model, ARIMA is fitted to time series data for better understanding of the data or predicting upcoming points in these series. Alzahrani et al. [14] have used ARIMA model to predict the estimated daily amounts of COVID-19 persons in Saudi Arabia. They have reported the superiority of ARIMA to Autoregressive Model, Moving Average and an integration ARMA and ARIMA. Using ARIMA, they have reported RMSE, MAE, R^2^ and MAPE values as 21.17, 14.93, 0.99 and 2.16, respectively [14]. Chakraborty et al. [15] have proposed a hybrid strategy founded on ARIMA model and Wavelet-based predictive model which could produce short-term predictions of the amount of daily cases for Canada, France, India, South Korea, and the UK. The obtained RMSE and MAE values ranged from 55.25-631.91 and 24–306.78 in different regions [15]. Khan et al. [16] have used an ARIMA model for forecasting daily cases of COVID-19 in India. They selected the appropriate model according to the Bayesian Information Criteria parameters and the total maximum R^2^ value of 0.95 [16]. The best performance measurements using ARIMA has been reported in the study conducted by Adiga et al. (MAPE = 999.1) [17].

### 3.3 Multilayer perceptron (MLP)

MLP is a type of feedforward artificial neural network (ANN). This model has three layers of nodes, namely an input layer, a hidden layer and an output layer. With the exception of the input node, other nodes are neurons that use a nonlinear activation function. MLP uses the backpropagation supervised learning method for training [18]. Car et al. [5] have used a freely accessible time-series dataset for design of their model. They have used this dataset in training an MLP model. The finest designed models had 4 hidden layers with 4 neurons in each. This model had appropriate measures in the prediction of the deceased and confirmed cases, but it had low robustness for recovered patients [5]. Pinter et al. [19] have used the hybrid machine learning strategies of ANFIS and MLP-imperialist competitive algorithm (MLP-ICA) for prediction of time series of COVID-19 cases and mortality amount. Short-term observation has confirmed the accuracy of the proposed model. Authors have suggested that the model keeps its exactness providing no substantial interruption happens [19].

### 3.4 Long short-term memory (LSTM)

LSTM is an artificial recurrent neural network (RNN) method utilized as a deep learning strategy. In contrast to standard feedforward neural networks, this model ensures feedback connection. In addition to processing single data points, LSTM can process complete sequences of data [20]. Aora et al. [21] have used RNN-related LSTM variants on an Indian dataset of COVID-19 patients to forecast the amount of positive cases. Based on the lowest error rate, LSTM model was selected for prediction of daily and weekly new COVID-19 cases with approximate error rates of 3% and 8%, respectively. Subsequently, they classified Indian states into different zones based on the extent of positive cases and daily escalation for recognition of COVID-19 hot-spots [21]. Fokas et al. [21] have applied a bidirectional LSTM network to yield a robust generalization of RNNs. This method has been used for predication of new cases of COVID-19 in Italy, Spain, France, Germany, USA and Sweden [22].

### 3.5 Other models

Yadav et al. [23] have used six regression analysis based methods including quadratic, third degree, fourth degree, fifth degree, sixth degree, and exponential polynomial for prediction of COVID-19 cases with the sixth degree polynomial regression method representing the best model for prediction of short-term new cases [23]. Kim et al. [24] have used geographic hierarchy to create Hi-COVIDNet according to a neural network with two-level machineries that are based on data collected from country-level and continent-level systems. This method apprehends the multifaceted relations among distant countries and relates their particular infection risk to the target country [24]. Table S1 shows the application of machine learning methods for prediction of COVID-19 spread.

## 4 Discussion

### 4.1 Synthesis of results

Accurate prediction of the time of outbreak would help in reduction of the effect of COVID-19, permit governments to modify their preventive strategies and plan in advance for the protective steps required. Modeling of COVID-19 spread is particularly important in defining its potential future impacts. Artificial intelligence methods are superior to traditional statistical modeling methods in the terms of offering high-quality predictive models [89]. These models are capable of identification of learning parameters that affect dissimilarities in COVID-19 spread across various regions or populations, combining numerous intervention methods and implementing what-if scenarios by integrating data from diseases having analogous trends with COVID-19. In the current scoping review, we compared the performance of several machine learning methods in prediction of COVID-19 spread. RMSE, MAE, R^2^ and MAPE parameters were selected as performance measures for comparison of the accuracy of models. R^2^ values have ranged from 0.64 to 1 for ANN and Bidirectional LSTM, respectively. ANFIS, ARIMA and MLP have also have R^2^ values near 1. ARIMA and LSTM had the highest MAPE values. These prediction models could also appraise the impact of climate-associated factors in infection rate or COVID-19 spread facilitating implementation of specific strategies for each condition. Moreover, the data obtained from these models can be used for categorization of county regions and identification of hot spots for COVID-19 to organize region-specific preventive measures. Incorporation of data from health status of affected individuals including general health situation and related risk factors would enhance the accuracy of these models. Most of the proposed models have been effective in short-term forecasting of the COVID-19-related parameters. However, their efficacy in long-term should be validated in further studies.

Modeling of the COVID-19 is practically important in defining the possible upcoming impact of this disorder and artificial intelligence methods have especial situation in this regard. These modeling strategies have implications in disease management by policy makers as they can predict the future course of the pandemic. Moreover, the impact of large-scale screening strategies and application of disease-controlling modalities can be considered in these modeling methods. ARIMA and LSTM have good performance values in this regard. In fact, ARIMA model is the furthermost extensively used forecasting method for prediction of trends in time series. However, it is not possible to compare the results of these studies, as these methods have not been applied and trained on the same data. Moreover, although artificial intelligence strategies have been promising in prediction of COVID-19 course during the pandemics, COVID-19 continues to be an unknown disease with no historic information to predict its spreading. Therefore, integration of these methods and implementation of the results in larger populations consisting of different ethnicities would help in design of better predictive models.

ARIMA method of time has been used to predict the stability and growth of COVID-19. Recent studies have suggested that the performance of this model can be enhanced or the model can provide more precise data if more numbers of datasets are accessible [90]. The model provides results according to the data established by information provided by health organizations. Therefore, prediction may not be completely precise, yet it can confidently be used as a corrective tool [90]. Combination of new factors and algorithms with ARIMA can lead of enhancement of accuracy.

Accordingly, Abbasimehr and Paki have proposed three hybrid methods for prediction of COVID-19 time series methods according to conjoining three deep learning models, namely multi-head attention, LSTM, and CNN with the Bayesian optimization algorithm. Their analyses have shown higher performance of deep learning models compared with the benchmark model both for short-term prediction and long-term prediction. Particularly, the mean SMAPE of the best deep learning model has 0.25 and 2.59 for the short-term and long-term predictions, respectively [25].

Deep Neural Networks (DNNs) has also been suggested as method for approximation. This method is an important alternative to estimate the solution of a Partial Differential Equation [91]. DNN has been used for detection of COVID-19 based on CT scan and chest X rays [92]. Application of unsupervised learning methods in which algorithm training is achieved using unlabeled data is another approach which is less studies in this context. A recent study has used the k-means algorithm to divide the countries into clusters based on the spread of COVID-19 in three time spans [93].

### 4.2 Summary of evidence

These forecasts are just built on past trends of COVID-19 spread, so forecast values are not definite. Nevertheless, these predicted estimates of events can assist authorities to establish resource planning for better management of this pandemic. Moreover, these methods can be used for prediction of need for preventive measures in each geographical region, thus helping vaccine manufacturers for designing appropriate plans.

### 4.3 Limitations

Impossibility of accurate comparison of methods, lack of consistency between study variables.

## Declarations

### Author contribution statement

All authors listed have significantly contributed to the development and the writing of this article.

### Funding statement

This study was financially supported by 10.13039/501100005851Shahid Beheshti University of Medical Sciences.

### Data availability statement

Data will be made available on request.

### Declaration of interests statement

The authors declare no conflict of interest.

### Additional information

No additional information is available for this paper.

## Figures and Tables

**Table S1:** Summary of the results of studies which used machine learning methods for prediction of COVID-19 spread.

Author, Year	Region(s)	Data Source	Data Structure	Best Algorithm/Model Structure(s)	Performance Measurements (on the best model)				Ref
					RMSE	MAE	R^2^	MAPE	
Abbasimehr, H., et al. 2021	Dataset 1: US, United Kingdom, Turkey, Spain, Mexico, Italy, Iran, Germany, France, Belgium	Humanitarian Data Exchange (Dataset 1 and 2 were used for short-term and long-term prediction, respectively)	Daily confirmed cases from 20 January to 1 August 2020	Attention-based model using Bayesian Optimizer	2715.12	-	-	0.2157	[25]
	Dataset 2: US, Brazil, India, Russia, South Africa, Mexico, Peru, Chile, Colombia, Iran		Daily confirmed cases from 20 January to 3 August 2020	LSTM using Bayesian Optimizer	25292.337	-	-	2.6606	
Adiga, A., et al. 2021	Maricopa AZ	Johns Hopkins University Center for Systems Science and Engineering dataset	The 7-day smoothed version of confirmed cases	SEIR (not machine learning)	-	-	-	1576.1	[17]
	Los Angeles CA			Spatial Autoregressive				1678.7	
	San Bernardino CA			ARIMA				999.1	
	Kings NY			LSTM				2085.4	
Al-Qaness, M. A. A., 2021	Brazil	Official WHO data	Daily confirmed cases from 26 March to 1 June 2020	MPA + ANFIS	19,432	14,273	-	0.3117	[26]
	Russia				493	379		0.03223	
Al-Qaness, M., A., A., et al., 2020	Korea	WHO website	Daily confirmed cases in the USA, Korea, Iran, and Italy from 22 January to 7 April 2020	MPA-ANFIS	70.93	60.31	96.48%	0.696	[10]
	Italy				5465.66	3951.94	98.59%	2.734	
	Iran				302.37	217.27	98.74%	0.736	
	USA				15611	12,979	95.95%	5.74	
Al-Qaness, M., A., A., et al., 2020	China	WHO website	Daily confirmed cases from 21 January to 18 February 2020	FPASSA-ANFIS	5779	4271	0.9645	4.79	[10]
Alsayed, A., et al., 2020	Malaysia	Ministry of Health Malaysia	Daily confirmed cases from 22 March to 5 April 2020	ANFIS	46.87	-	0.9973	2.79	[12]
Alzahrani, S., et al., 2020	Saudi Arabia	Saudi Ministry of Health website	Daily confirmed cases in Saudi Arabia	ARIMA	21.17	14.93	0.99	2.16	[14]
Ardabili, S. F., et al., 2020	Italy	Worldometer website	Daily confirmed cases over 30 days	MLP	191.27	-	0.999	-	[27]
	Germany				55.52		0.995		
	Iran				391.8		0.998		
	USA				22.1		0.999		
	China				2318.22		0.999		
Arora, P., et al, 2020	India	Johns Hopkins University Center for Systems Science and Engineering dataset	Daily confirmed cases from 14 March to 14 May 2020	Stacked LSTM	3.22	-	-	-	[21]
Arora, P., et al. 2020	India	Ministry of Health and Family Welfare (Government of India)	State-wise daily confirmed cases from 14 March to 14 May 2020	Bi-directional LSTM	-	-	-	3.22	[21]
ArunKumar, K. E., et al. 2021a	USA	Johns Hopkins University Center for Systems Science and Engineering dataset	Cumulative daily confirmed cases	LSTM	1.69E + 06	-	-	-	[28]
	Brazil				1.33E + 05				
	South Africa				6.03E + 03				
	Peru				4.44E + 03				
	Chile				1.22E + 03				
	Iran				1.33E + 02				
	Mexico			GRU	4.00E + 03				
	UK				4.21E + 02				
	Russia				9.37E + 02				
	India				2.14E + 04				
ArunKumar, K. E., et al. 2021b	South Africa	Johns Hopkins University Center for Systems Science and Engineering dataset	Cumulative daily confirmed cases 22 January to 24 July 2020	Seasonal ARIMA	1.48E+04	1.17E+04	-	5.46	[29]
	Bangladesh				3.40E+03	2.92E+03		2.57	
	Brazil				2.34E+03	2.13E+03		3.12	
	Chile				9.07E+03	7.80E+03		2.53	
	Colombia				2.38E+02	1.16E+02		0.13	
	India				1.21E+04	7.15E+03		0.87	
	Iran				4.65E+03	3.80E+03		0.095	
	Italy				1.94E+03	1.79E+03		0.923	
	Mexico				1.09E+04	8.82E+03		3.24	
	Pakistan				3.07E+02	1.58E+02		0.078	
	Peru				2.41E+03	1.99E+03		0.77	
	Russia				1.40E+04	1.23E+04		97	
	Saudi Arabia				1.15E+04	1.08E+04		0.08	
	Spain				2.00E−03	2.00E−02		0.988	
	UK				1.45E+01	1.25E+01		0.80	
	USA				2.46E+04	1.72E+04		1.41	
Ayyoubzadeh, S. M., et al., 2020	Iran	Worldometer website and Google Trends	Customized dataset consisting of the daily incidence from 15 February, 2020, to 18 March, 2020 and Google search trends	Linear regression	7.562	-	-	-	[30]
Bedi, P., et al. 20201	India	covid19india.org website	Daily confirmed cases 30 January to 6 September 2020	LSTM	-	-	-	0.03	[31]
Behnood, A, et al, 2020	USA	USAFacts Website	Daily confirmed cases in 1657 counties	ANFIS-VOA-II	22.4744	7.3337	0.8339	-	[13]
Borghi, P. H., et al. 2021	Global (top 30 countries with the highest number of daily new cases)	Johns Hopkins University Center for Systems Science and Engineering dataset	Daily confirmed cases till 11 May 2020	ANN	2.082E+03	3.718E+06	-	-	[32]
Car, Z., et al., 2020	406 locations	Johns Hopkins University Center for Systems Science and Engineering dataset	Daily confirmed, recovered, and deceased patients in a certain location (defined by the name of location, latitude, and longitude), from 22 January to 12 March 2020	MLP	-	-	0.98599	-	[5]
Chakraborty, T., et al., 2020	USA	Our World in Data Website	Daily confirmed cases	TARNN	721.5658	468.6335	-	-	[33]
	Brazil				178.0458	90.2053			
	India				201.0696	128.7718			
	Russia				443.4280	202.6083			
	South Africa				243.5067	160.3598			
	Mexico				24.4335	15.1298			
	Spain				136.3910	87.5449			
	Iran				319.9160	182.8744			
Chakraborty, T., et al., 2020	Canada	Our World in Data Website	Daily confirmed cases	Hybrid ARIMA-WBF Model	149.60	40.05	-	-	[15]
	France				631.91	306.78			
	India				55.25	24.00			
	South Korea				90.29	54.06			
	UK				180.66	100.68			
Chatterjee, A., et al. 2020	China, Italy, Spain, Germany, Iran, Switzerland, South Korea, Belgium, Netherlands, Astria, Singapore, Malaysia, France, Australia, USA, UK and Portugal	Our World in Data Website and a simulated dataset	Daily confirmed, and deceased patients from 1 January to 2 April, 2020	Bidirectional LSTM	8,649.154	7,130.149	1	-	[34]
Chaurasia, V., et al., 2020	Worldwide	DataHub-Novel Coronavirus 2019-Dataset	Daily confirmed, recovered, and deceased patients from 22 January to 29 June, 2020	ARIMA	0.1517	0.12044	-	0.0091	[35]
Chimmula, V., K., R., et al., 2020	Canada	Johns Hopkins University and Canadian Health authority	Daily confirmed, recovered, and deceased cases from 22 January to 31 March, 2020	LSTM	34.83 (short-term predictions) 45.70 (long-term predictions)	-	-	-	[36]
Chowdhury, A. A., et al. 2021	Bangladesh	Worldometer website	Daily confirmed cases 10 April to 30 June 2020	LSTM	6.55	-	-	4.51	[37]
da Silva, C. C., et al. 2021	Brazil	Brasil.io portal	Daily confirmed cases till 6 June 2020	Linear Regression	11.42%	-	-	-	[38]
	Pernambuco (A state in brazil)				1.92%				
de Souza, D. G. S. et al., 2020	Amapa (A state in Brazil)	Health surveillance secretary of Amapa	Cumulative confirmed cases from 20 March to 31 August, 2020	Holt-Winters	162	-	0.98	0.34	[39]
Dharani, N. P., et al. 2021	India	Kaggle website	Daily confirmed cases 30 January to 21 May 2020	Linear Regression	223.89	157.78	1.0	-	[40]
Doe, S. W., et al., 2020	USA	Johns Hopkins University confirmed cases data for US counties	Daily confirmed cases from 22 January to 31 May, 2020 and latitude, and longitude of each county	CLEIR-Net	264.33	-	-	-	[41]
Fokas, A. S, et al., 2020	Italy	European CDC website	Daily confirmed cases	Bidirectional LSTM network	538	-	0.9999	-	[22]
	Spain				1022		0.9998		
	France				821		0.9997		
	Germany				1128		0.9997		
	USA				10754		0.9996		
	Sweden				178		0.9997		
Ganiny, S., et al., 2020	India	Worldometer website, India's Ministry of Health and Family Welfare, the Covindia website	Daily confirmed, recovered, and deceased cases from 1 March to 25 July, 2020	ARIMA	457.61	330.79	0.99998	0.2471	[42]
Ghany, K. K. A., et al. 2021	Saudi Arabia	Johns Hopkins University Center for Systems Science and Engineering dataset	Daily confirmed cases 22 January to 24 July 2020	LSTM	1768.35	-	-	-	[43]
	Qatar				735.21				
	Oman				730.53				
	Kuwait				456.90				
	UAE				446.44				
	Bahrain				320.79				
Ghazaly, N., M., et al., 2020	Egypt, Saudi Arabia, Jordan, USA, Spain, Italy, France, Iran, Russia	WHO situation reports	Daily cases and deaths from 21 January to 2 April, 2020	NAR	-	-	-	2.6521	[44]
Guo, Q. and He, Z., 2021	Global	Official WHO data	Daily confirmed cases 21 January to 11 November 2020	ANN	3102.9	2090.6	0.9683	-	[45]
Hasan, K. T., et al. 2021	Bangladesh	Official WHO data and the Institute of Epidemiology, Disease Control and Research of Bangladesh	Daily confirmed cases till 3 August 2020 + Government control and people's compliance data + Information of how many people will be in contact with an infected person outside of their home when they move out	LSTM	10,368.318	-	1	5.96	[46]
Hazarika, B. B., et al., 2020	Brazil	Our World in Data Website	Cumulative number of confirmed cases from 11 April to 10 July, 2020	WCRVFL (Using sigmoid or ReLu activation functions)	0.00323	-	0.99975	-	[47]
	India				0.00147		0.99996		
	Peru				0.00197		0.99986		
	Russia				0.00029		0.99999		
	USA				0.00524		0.99999		
Hawas, M, et al., 2020	Brazil	Johns Hopkins University Center for Systems Science and Engineering dataset	Daily cases from 22 January, 2020, to several dates	RNN	-	-	0.665	-	[48]
Heni¸ B., et al., 2020	Phase I: 79 countries phase II: China, South Korea, France, Germany, Iran, Iraq, United Kingdom, Italy, Japan, Singapore, Spain, Thailand	European CDC website	Phase I: Daily confirmed cases from the date of the first confirmed cases to 13 March, 2020 Phase II: Daily recovered cases from the date of the first confirmed cases to 19 March, 2020	LSTM	-	-	Phase I: 0.999 Phase II: 0.996	-	[49]
Hridoy, A. E., et al., 2020	Bangladesh	Johns Hopkins University's GitHub repository	Daily confirmed, recovered, and deceased cases from 8 March to 13 June, 2020	Stacked LSTM	593.764	-	0.95	1.76%	[50]
Kasilingam, D., et al. 2021	Global (42 countries)	Official WHO data, the World Bank website, the Weather Underground website	Daily confirmed cases 22 January to 24 March 2020 + infrastructure, environment, policies, and infection-related independent variables	Random forest	-	-	0.543 to 0.992 (country-wise)	-	[51]
Kırbas¸ I., et al., 2020	Denmark	European CDC	Daily confirmed cases from the date of the first confirmed cases to 3 May 2020	LSTM	54.5398	-	0.999963324	0.5033	[52]
	Belgium				274.0248		0.999967249	0.5422	
	Germany				569.4791		0.999987029	0.3083	
	France				455.7141		0.999987339	0.3155	
	United Kigdom				5482.2361		0.998923082	2.5025	
	Finland				49.4966		0.999897188	0.8492	
	Switzerland				55.8685		0.999996376	0.1640	
	Turkey				640.26257		0.999971407	0.4823	
Khajanchi, S., et al., 2020	Jharkhand	COVID19 INDIA Website	Daily confirmed cases and the cumulative confirmed cases up to 24 May, 2020	SAIUQR	5.23	-	-	-	[53]
	Gujarat				51.82				
	Andhra Pradesh				13.47				
	Chandigarh				3.82				
Kim, M., et al., 2020	South Korea	Johns Hopkins University Center for Systems Science and Engineering dataset, Google trends, International Roaming (by Korea Telecom), airline information system, Korean CDC	Daily confirmed cases + Searched keyword (“COVID-19,” “COVID test,” “Flu,” “Mask”) + Inter-Country Data including customers and airlines arriving in Korea, imported cases in Korea	Hi-COVIDNet (A Customized RNN using LSTM layers)	May 6–12: 0.4373 May 6–19: 0.4045	-	-	-	[24]
Khan, F., et al., 2020	India	Ministry of Health and Family Welfare, COVID19 INDIA Website	Daily confirmed cases up to 4 April, 2020	ARIMA	-	-	0.95	-	[16]
Kufel, T., et al., 2020	32 European countries	Johns Hopkins University Center for Systems Science and Engineering dataset	Daily confirmed, recovered, and deceased cases for 14 days in each country	ARIMA	-	-	From 0.5577 to 1.0000 (depends on country and dates)	From 0.0228 to 83.3660 (depends on country and dates)	[54]
Kumar, S., et al., 2020	India	COVID19 INDIA Website	Cumulative number of confirmed, recovered, and deceased cases	ARIMIA	641.732 (new cases)	-	0.987 (new cases)	-	[55]
					705.293		1.000 (total cases)		
Kumar, N., et al., 2020	Worldwide	Johns Hopkins University Center for Systems Science and Engineering dataset	Daily confirmed, recovered, and deceased cases up to 20 May, 2020	ARIMA	36992.53	34932.99	-	2.523	[56]
	US				33109.68	31899.89		15.635	
	Spain				9774.06	9683.45		7.361	
	Italy				13078.23	12910.06		12.78	
	France				5853.29	5780.87		10.574	
	Germany				13901.04	13702.61		9.808	
	Russia				3212.50	2376.69		5.103	
	Iran				4496.75	4213.14		4.933	
	UK				91.12	78.19		8.311	
	Turkey				4333.57	4242.09		4.321	
	India				1066.65	721.17		2.911	
Liu, Z., et al., 2020	Wuhan, Beijing, Shanghai, Guangzhou	Tencent news and Baidu migration websites	Daily confirmed cases	ANN	-	-	0.9969	-	[57]
Majhi, R., et al. 2020	China (for training), India (for validation)	*NA*	Daily confirmed and recovered cases, daily deaths, Amount of testing, Lockdown presence and its severity	Random forest	-	-	-	0.02	[58]
Malki, Z., et al. 2021	Worldwide	Johns Hopkins University, WHO and Worldometer official website	Daily confirmed cases	Decision Tree	0.085	0.047	0.993	0.160	[59]
	USA				0.068	0.049	0.995	0.107	
	Brazil				0.106	0.058	0.989	0.073	
	India				0.073	0.050	0.995	0.248	
	Spain				0.152	0.098	0.977	0.207	
	Italy				0.062	0.038	0.996	0.113	
	France				0.133	0.069	0.982	0.277	
	UK				0.075	0.044	0.994	0.126	
	Germany				0.060	0.040	0.996	0.050	
	Russia				0.094	0.055	0.991	0.308	
	Turkey				0.065	0.033	0.996	0.051	
Mishra, P., et al., 2020	India	WHO daily situation reports	Daily new cases from 17 March to 1 July, 2020	ANN	38.22	23.12	-	-	[60]
Moftakhar, L., et al., 2020	Iran	Iran Ministry of Health and open datasets provided by Johns Hopkins University	Daily new cases from 19 February to 30 March 2020	ARIMA	1539.43	24.85	-	-	[61]
Melin, P, et al., 2020	Mexico	Government of Mexico website	Daily confirmed and deceased cases	Modular Neural Network with Fuzzy	From 8.6153 to 1554.0302 (Depends on the state)	-	-	-	[62]
Mollalo, A. et al. 2020	USA	USAFacts website	Cumulative number of confirmed cases from 22 January to 30 April 2020	ANN	0.722409	0.355843	0.645481	-	[63]
Nabi, K. N., et al. 2021	Brazil	Johns Hopkins University Center for Systems Science and Engineering dataset	Daily confirmed cases till 18 November 2020	CNN	0.086	-	-	6.94	[64]
	Russia				0.014			0.85	
	UK				0.048			3.75	
Neeraj, et al., 2020	Canada	Johns Hopkins University Center for Systems Science and Engineering dataset	Daily confirmed, recovered, and deceased cases for 209 days in each country	Attention-based encoder-decoder	12.46	-	-	0.11	[65]
	Italy				209.23			1.71	
	France				163.78			1.21	
	Spain				281.03			2.11	
Nikolopoulos, K, et al., 2020	Germany, India, Singapore, UK, USA	Johns Hopkins university dataset, “covid19-report” website, Mayer Brown's COVID-19 Global Travel Restrictions, the world population review, World Life Expectancy website, World Bank website	Daily confirmed, recovered and deceased cases, climate information, travel restrictions and curfews data, populations information, lung diseases data, coronary heart diseases data, diabetes prevalence data, GDP spent on healthcare data	Naive-d 0.1 for weekly prediction	-	1.0015 (Scaled)	-	1.0022	[66]
				GARCH(1,1) model with SGED for daily prediction		0.2064 (Scaled)		0.2160	
Pal, R., et al., 2020	USA	Johns Hopkins university dataset, Dark Sky website	Daily confirmed, recovered and deceased cases from 22 January to 2 August, 2020 and weather data	Shallow LSTM using used a Bayesian optimization framework	1103.5	-	-	-	[67]
Papastefanopoulos, V., et al., 2020	US	Novel Corona Virus 2019 Dataset and population-by-country dataset on Kaggle website	Daily confirmed, recovered and deceased cases as of 4 May 2020	TBAT	0.009873	-	-	-	[68]
	Spain				0.029295				
	Italy				0.005810				
	UK				0.004310				
	France				0.007003				
	Germany				0.003389				
	Russa				0.002193				
	Turky				0.001946				
	Brazil				0.005621				
	Iran				0.000425				
Peng, Y., et al. 2021	Worldwide (215 countries)	Official WHO data, Google Trends service	Daily confirmed cases 10 January to 16 August 2020 + Infoveillance data (Google Trends (search volume of 28 COVID19-related features))	Random forest	9.27	5.42	-	-	[69]
Pereira, I. G., et al., 2020	USA, Brazil, Italy, Spain, France, UK	Johns Hopkins university dataset, Natalnet's Lab and Brazil ministry of health (just for Brazil), Italy—Official Covid Data Repository.	Daily confirmed and deceased cases	LSTM-SAE	-	-	0.822	84	[70]
Perone, G., 2020	Italy	Worldometer website	Daily confirmed cases (Italy (February 22–April 14), USA (March 9–May 16), Russia (March 22–May 22))	ARIMA	412.79	283.49	0.95	13.039	[71]
	Russia				606.66	430.83	0.98	11.39	
	USA				2,411.6	1,631.3	0.95	9.59	
Perone, G., 2020	Italy	Italian Ministry of Health's website	Daily confirmed cases from 21 February to 13 October, 2020	ARIMA-NNAR	190.542	106.4554	-	2.1901	[72]
Pinter, G., et al., 2020	Hungary	Worldometer website	Daily confirmed, and deceased cases from 4 March to 19 April, 2020	MPL-ICA	167.88	-	-	-	[19]
Quintero, Y., et al. 2021	Colombia	The National Institute of Health for Colombia and the National Administrative Department of Statistics	Daily confirmed cases from March to July 2020 + Socioeconomic data including people over 65, poverty index, total population, people per km^2^, Average age, average morbidity	Gradient boosting regressor	0.0157	0.0045	0.8986	1.5317	[73]
Ribeiro, Mhdm, et al., 2020	Brazil	WHO website	Daily confirmed, and deceased cases from 15 March to 19 April, 2020	SVR (generally was the best algorithm, stacking-ensemble learning and ARIMA outperformed in some cases)	-	18–409 (One day) 8.5–59.67 (Three days) 7.83–73.17 (Six days)	-	0.87–3.51 (One day) 1.02–5.63 (Three days) 0.95–6.90 (Six days)	[74]
Rustam, F., et al., 2020	Afghanistan, Australia, Algeria, Canada	Johns Hopkins University Center for Systems Science and Engineering dataset	Daily confirmed, recovered, and deceased patients	Exponential Smoothing	16828.58	8867.43	0.98	-	[75]
Saba, A. I., et al., 2020	Egypt	The Egyptian Ministry of Health	Accumulated confirmed cases from 1 March to 10 May, 2020	Nonlinear Autoregressive ANN	10.410	7.752	0.999	-	[76]
Said, A. B., et al. 2021	Worldwide data	Official WHO data	Daily confirmed cases + Demographic, socioeconomic, and health sector indicators data	Bidirectional LSTM	245.1	176.02	0.996	-	[77]
Saqib, M., 2020	US	Johns Hopkins University Center for Systems Science and Engineering dataset	Daily confirmed patients	Hybrid polynomial-Bayesian ridge regression model	723.75	-	-	-	[78]
	Italy				418.36				
	Spain				624.27				
Senapati, A., et al. 2021	Assam (a state in India)	Kaggle website	Daily confirmed cases till 30 October 2020	Piecewise linear regression	-	-	-	0.392	[79]
Shyam Sunder Reddy, K., et al. 2020	India	“Our World in Data” website	Daily confirmed cases 15 February to 18 September 2020	LSTM	0.767	-	-	-	[80]
Tabar, B. R., et al., 2020	East Midlands region of England	Public Health UK and NHS Digital	Daily cumulative confirmed cases and the total number of daily phone calls received at the NHS 111 from 18 March to 19 September, 2020	MLR-T	19.37	14.16	-	-	[81]
Torrealba-Rodriguez, O. et al., 2020	Mexico	Daily Technical Report" by the Mexican Ministry of Health	Daily confirmed cases from 27 February to 8 May, 2020	ANN	-	-	0.9999	-	[82]
Tuli, S., et al., 2020	World	Our World in Data website	Daily confirmed cases	Generalized Inverse Weibull distribution fitting	-	-	0.98	49.14	[83]
	India						0.97	18.33	
	USA						0.95	24.33	
	UK						0.95	21.46	
	Italy						0.96	14.98	
Tuli, S., et al., 2020	World	Our World in Data website, Index Mundi, World Bank, Oxford Government Response Tracker	Daily confirmed cases till 19 May, 2020, Socioeconomic data, Virus Type data, Government Stringency Index	LSTM-based Robust Weibull approach	559.46	-	0.93	39.29	[84]
	India				4.39		0.91	22.00	
	USA				319.37		0.86	26.09	
	UK				99.60		0.90	14.93	
	Italy				45.93		0.89	12.78	
Wang, L., et al., 2020	Global (Austria, Brazil, India, Italy, Nigeria, Singapore, the United Kingdom)	UVA COVID-19 surveillance dashboard, Johns Hopkins University dataset, Google COVID-19 Aggregated Mobility Research dataset	Weekly confirmed and deceased cases, Case count growth rate, COVID-19 testing data, aggregated relative weekly mobility flows over google users, Flow Reduction Rate of connectivity before and after the pandemic, and Social Distancing Index, All from 7 March to 22 August, 2020 (25 weeks)	Ensemble of RNNs and SEIR	Was 37 at minimum (depends on the algorithm, data, and the region)	-	-	Was 12 at minimum (depends on the algorithm, data, and the region)	[85]
	US-States								
	US-Counties								
Yadav, R. S., 2020	India	COVID-19 in India Kaggle dataset	Daily confirmed cases from 1 March to 11 April, 2020	Sixth degree polynomial regression analysis	-	-	0.9990	-	[23]
Zawbaa, H. M., et al. 2021	China	Johns Hopkins University Center for Systems Science and Engineering dataset and European Centre for Disease Prevention and Control	Daily confirmed cases 22 January to 13 December 2020	ANN	1460.57	-	-	-	[86]
	Cote d’Ivoire				105.94				
	Kenya				486.13				
	Egypt				318.29				
	Algeria				371.40				
	Japan				952.29				
	Iran				5632.84				
	Italy				17922.58				
	USA				77822.38				
Zeroual, A., et al. 2020	Italy	Johns Hopkins University Center for Systems Science and Engineering dataset	Daily confirmed cases till 17 June 2020	Bidirectional LSTM	1,041,374	1,033,467	-	4398	[87]
	Spain				1,194,711	1,187,629		4916	
	France			LSTM	1,085,008	1,075,795		5738	
	USA				1,129,183	1,123,909		58,008	
	China			Variational Auto-encoder	11,103	107,873		128	
	Australia				18,732	17,186		236	
Zeroual, A., et al., 2020	Italy	Johns Hopkins University Center for Systems Science and Engineering dataset	Daily confirmed, recovered, and deceased cases from 22 January to 17 June, 2020	VAE	1,386,225	1,385,829	-	5.90	[87]
	Spain				5,315,748	5,288,172		2.19	
	France				3,688,083	3,522,353		1.88	
	China				11,103	107,873		0.128	
	Australia				18,732	17,186		0.236	
	USA				4,079,244	3,976,682		2.04	
Zheng, N., et al., 2020	Wuhan	Private dataset	Customized dataset consist of the daily cases which are confirmed, suspected, cured, died and social and news media	A hybrid AI system through improved susceptible–infected model, NLP and LSTM	-	239.83	-	0.52	[6]
	Beijing					0.50		0.38	
	Shanghai					0.17		0.05	
	Countrywide (China)					659.00		0.86	
Zivkovic, M., et al. 2021	China	Official WHO data	Daily confirmed cases 21 January to 18 February 2020 + 10 November to 10 December 2020	Cauchy exploration strategy BAS + ANFIS	4329	3195	0.9763	4.08	[88]
		“Our World in Data” website			4106	2994	0.9775	4.08	

(Regions: Regions that model was evaluated in, Data Source: The source(s) that data were acquired from, Data Structure and Size: Data modalities and dates of data collection, Best Model Structure(s): Best machine algorithm or deep learning model reported in the selected paper based on its performance, Performance Measurements (on the best model): The measurement of the model output performance based on RMSE, MAE, R2 value, and MAPE).
